# High-Reynolds Microfluidic Sorting of Large Yeast Populations

**DOI:** 10.1038/s41598-018-31726-6

**Published:** 2018-09-13

**Authors:** Eliezer Keinan, Ayelet Chen Abraham, Aaron Cohen, Alexander I. Alexandrov, Reshef Mintz, Merav Cohen, Dana Reichmann, Daniel Kaganovich, Yaakov Nahmias

**Affiliations:** 1 Alexander Grass Center for Bioengineering, Benin School of Computer Science and Engineering, The Hebrew University of Jerusalem, Jerusalem, Israel; 2 Department of Cell and Developmental Biology, Silberman Institute of Life Sciences, The Hebrew University of Jerusalem, Jerusalem, Israel; 3 Department of Biological Chemistry, Silberman Institute of Life Sciences, The Hebrew University of Jerusalem, Jerusalem, Israel; 4 Present Address: Fundamentals of Biotechnology Federal Research Center of the Russian Academy of Sciences, Bach Institute of Biochemistry RAS, Moscow, Russia; 5 Present Address: Department of Experimental Neurodegeneration, UniversityMedical Center Goettingen, Goettingen, Germany

## Abstract

Microfluidic sorting offers a unique ability to isolate large numbers of cells for bulk proteomic or metabolomics studies but is currently limited by low throughput and persistent clogging at low flow rates. Recently we uncovered the physical principles governing the inertial focusing of particles in high-Reynolds numbers. Here, we superimpose high Reynolds inertial focusing on Dean vortices, to rapidly isolate large quantities of young and adult yeast from mixed populations at a rate of 10^7^ cells/min/channel. Using a new algorithm to rapidly quantify budding scars in isolated yeast populations and system-wide proteomic analysis, we demonstrate that protein quality control and expression of established yeast aging markers such as CalM, RPL5, and SAM1 may change after the very first replication events, rather than later in the aging process as previously thought. Our technique enables the large-scale isolation of microorganisms based on minute differences in size (±1.5 μm), a feat unmatched by other technologies.

## Introduction

**Figure 1:**
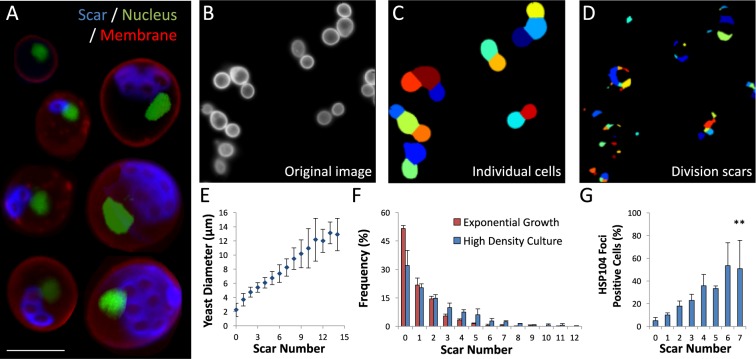
Single cell characterization of replicative aging in yeast (**A**) Combined image set of single yeast cells showing 0 to 10 budding scars. Chitin scars are stained with calcofluor white (blue), membranes with rhodamine-concanavalin A (red), and nuclei tagged with Htb2-GFP (green). Bar = 5 μm. **(B)** Image of rhodamine-concanavalin A stained yeast cells. **(C)** Single yeast cells identified using our algorithm. Objects touching image borders were discarded. **(D)** Budding scars identified using our algorithm. **(E)** Quantification of average membrane diameter as a function of scar number calculated from over 5000 yeast cells using our algorithm. Yeast shows a linear growth rate of 0.8 ± 0.1 μm/scar, and clear overlap between age groups. **(F)** Replicative age distribution in an exponential growth culture and in a high-density culture (*methods*). **(G)** Percent of yeast with GFP-tagged HSP104 foci as function of replicative age, calculated using image processing of 930 cells. **p < 0.01.

*Saccharomyces cerevisiae*, the budding yeast, is an important model for the molecular study of cellular aging^1^. Yeast undergo asymmetric aging by partitioning aging factors, including damaged proteins, organelles, and membrane components, away from an emerging daughter cell that is rendered pristine^2^. Dissecting the mechanism of asymmetric divisions in yeast can thus shed light on mammalian asymmetric aging and age-related pathologies^3^. Interestingly, as yeast age, they accumulate chitin-rich bud scars that are easily detectable using calcofluor white staining, enabling precise scoring of their replicative age (Fig. 1A). Yeast cells also increase in size with every division starting at 2 μm diameter as a virgin bud and growing up to 10–20 μm diameter as the cells age. However, the tiny fraction of adult yeast present at any given time, limits single cell analysis. This limits our ability to study the degradation rates of misfolded proteins, or to carry out proteomic and metabolomic-type analysis that require large quantities of aging cells.

Current methods to isolate yeast populations, including mother cell enrichment^4^, elutriation^5^, and single-cell micro-trapping^6,7^, cannot easily generate high quantities of adult cells as they rely on batch and low-throughput processes. Age synchronization of large quantity of yeast was demonstrated with binding of iron beads to biotinylated cells and the capture of an entire culture at its exponential phase, using a magnetic field^8^. This method was recently applied to characterize age dependent determinants of a synchronized culture at different time points during culture^9^. The inspection of yeast begins only 7.8 hours after capture in this method to avoid stress response and it is insensitive to replicative age heterogeneity within the captured population. To reveal earlier aging processes, higher resolution sorting of cells from the same culture is required.

One approach for continuous high throughput isolation of aging yeast utilizes microfluidic inertial focusing. Inertial focusing is the trapping of particles via opposing lift forces acting perpendicular to the direction of flow^10^. Using this approach, Di Carlo and colleagues were able to focus large particles while leaving small particles homogeneously distributed^10^, while others used Dean drag forces to segregate small and large particles to different streamlines^11^. Both of these methods are unable to separate similarly sized particles with high resolution. For this reason, recent attempts to separate yeast populations were limited to separating attached mother-daughter pairs from virgin buds where the size difference is greatest^12^. Another drawback of existing inertial focusing methods is the slow flow rate that is typically used in microfluidic devices, limiting throughput and preventing continuous operation due to repeated clogging^13^.

Recently, we showed that large particles are pushed to the concave edge of curved channels in high-Reynolds flow due to opposing shear-induced lift forces^14^. The phenomenon permits a 100-fold miniaturization of microfluidic devices, operating at high flow rates thereby eliminating clogging while increasing throughput. In this work, we combine this high-Reynolds inertial focusing mechanism with the preferential rejection of small-dense particles from peripheral Dean vortices, forcing small particles to the center of the channel. Using a single device we are able to isolate 10^7^ yeast/min from standard yeast suspensions. Together with a newly developed algorithm to quantify bud scars and cell diameter, we demonstrated that diminished protein quality control is observed already after 2–3 budding events, much earlier than previously reported^15^. Mass spectrometry of the sorted yeast populations revealed that the proteomic profiles of young and adult cell populations, originating from the same culture, significantly differ in expression of youthfulness and aging markers.

## Results

### Single cell size characterization of yeast

Budding yeast divide asymmetrically, producing a smaller daughter cell and a bud scar on the mother (Fig. 1A). Division is asymmetric, resulting in the accumulation of aging factors by the mother cell, while the daughter is rendered pristine^2^. To rapidly characterize yeast populations, we created an algorithm using CellProfiler^16^ that uses images of calcofluor white stained yeast (Fig. 1B) to identify single cells and quantify their diameter and scar count (Fig. 1C,D). Following experimental validation of our automated algorithm, we analyzed over 5,000 yeast cells demonstrating a linear increase in yeast diameter starting from 2.3 ± 1 μm for a virgin bud (0 scars) and plateauing around 12.2 ± 2 μm at 11 scars (Fig. 1E). Yeast diameter growth rate was 0.8 ± 0.1 μm/scar, showing significant overlap between age groups. To verify our membrane and scar detection, we analyzed populations of yeast in the exponential growth phase (Fig. 1F), of which 51.5 ± 1.5% of the yeast are newborn. These results confirm and reiterate previous measurements^17^ showing that replicative age distribution among the exponential phase population correlates well with yeast generation time, for yeast with 10 scars or less^18^. Analysis of yeast cells grown in high-density culture for several days showed that 32 ± 8% of the overall population was newborn, 67 ± 8% of the overall population had 0 to 2 scars, and 33 ± 8% had 3 or more scars (Fig. 1F). Less than 0.5% of the population had 11 scars or more. The different age distribution in the high-density culture suggests that the generation time homogeneity among adult yeast (<10 scars) is not maintained due to age dependent stress response. Interestingly, our analysis suggests that HSP104 inclusion increased gradually from the first steps of replicative aging (Fig. 1G). CellProfiler code is available upon request.

### Microfluidic size-dependent sorting in high flow rates

**Figure 2:**
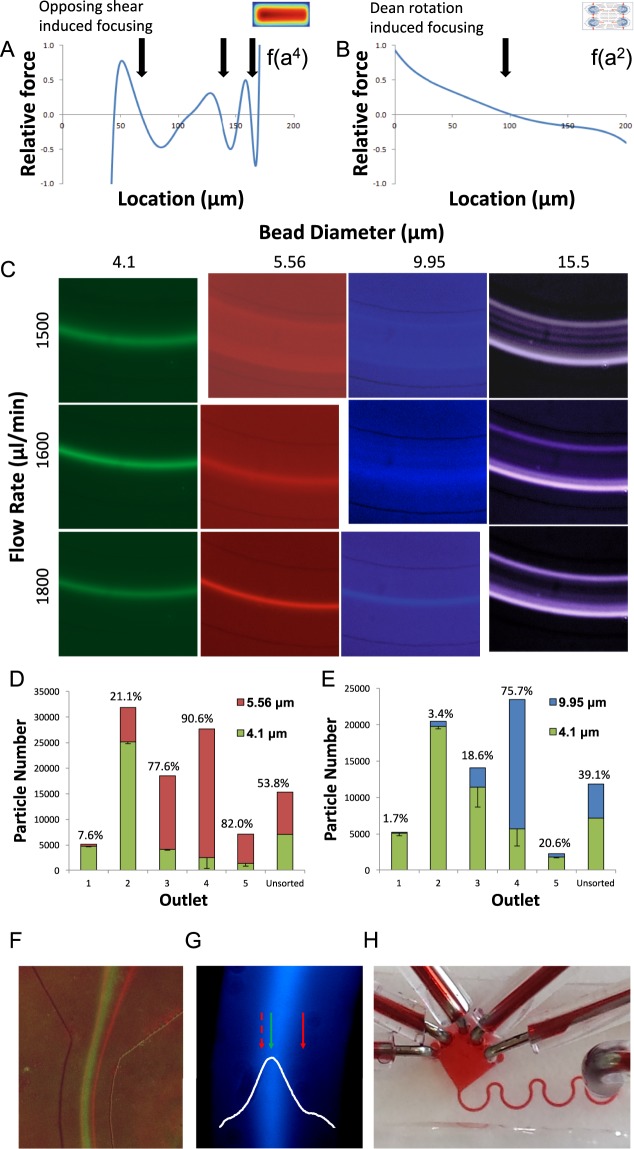
Microfluidic high Reynolds inertial sorting (**A**) Analytical calculation of shear-induced lift forces acting on 15 μm particle, in a horizontal cross-section of a rectangular channel, as previously shown^14^. Stable equilibrium points are indicated by black arrows. Numerically derived velocity profile is superimposed. **(B)** Illustration of centrifugal forces acting on a dense 4 μm particle exposed to two Dean vortices. Stable equilibrium point indicated by black arrows. **(C)** Long-exposure images of fluorescent beads focused in curved microfluidic channels with 0.9 mm radius of curvature. Inertia focusing predominates in large particles, pushing beads to the concave side of the channel, while Dean vortices traps small particles in the center of the channel. Particle size threshold for inertial focusing increases with fluid velocity. **(D,E)** Cytometry-based particle analysis of microfluidic outflow compared to unsorted particle mixture. Particles were isolated at 1.5 mL/min flow rate (Re = 215). High purity of small 4.1 μm particles was obtained at convex side (outlet 1) but at low 12 ± 1% yield. Small 4.1 μm particles were primarily focused by Dean vortices to the channel center (outlet 2) with a yield of 55 ± 5%. Purity of 4.1 μm ranged from 79 ± 1% compared to similarly sized 5.56 μm particles, with overlapping distribution (**D**) to 97 ± 1% when separated from larger 9.95 μm particles (**E**). Large 5.56 and 9.95 μm particles were primarily pushed toward the concave side of the channel (outlet 4) by opposing shear-induced forces. Purity of larger particles ranged from 76 ± 9% to 90 ± 5% for 9.95 μm and 5.56 μm particles, respectively, with similar yields of 60 ± 9%. **(F)** Long exposure image of 4.1 μm (green) and 5.56 μm (red) particle streaklines. The green particles are focused to the center of the channel, and red particles are primarily pushed toward the concave edge of the channel. **(G)** Long exposure image of a heterogeneous yeast sample (blue). The streakline’s highest intensity location (white histogram) coincides with the small bead streakline (4.1 μm, green arrow) rather than the large bead streakline (5.56 μm, red arrows). **(H)** Photo of the microfluidic device composed of 4 repeats of curved microfluidic channels.

Recently we showed that opposing shear-induced forces push large particles toward the concave side of curved channels in high flow rates (Fig. 2A). This force increases as a function of particle diameter to the 4^th^ power and predominantly works on larger particles^10^. Interestingly, a second lift force appears in curved channels due to the Dean vortices induced rotation (Fig. 2B). We note that particles will be pulled to the vortex center, due to higher particle velocity relative to the fluid in the inner side of the spinning particle^19^. Thus, a stable trap dominates at the center of the channel (Fig. 2B, *arrow*). These forces increase as a function of particle diameter to the 2^nd^ power^20,21^, and thus predominantly work on smaller particles.

To demonstrate our separation principle, we introduced fluorescent polystyrene beads to microfluidic devices containing repeating curves of 0.9 mm radius of curvature (*methods*). Bead diameter ranged from 4.1 to 15.5 µm while flow rates ranged from 1500 to 1800 μl/min, corresponding to Reynolds of 215 to 257 (Fig. 2C). We show that small particles are predominantly focused to a single streamline at the center of the channel due to Dean vortices induced lift force, while large particles are forced to the concave edge of the channel due to opposing shear-induced forces. Interestingly, increasing fluid velocity changes the threshold between focusing mechanisms, rejecting larger particles from the Dean vortices toward the center (Fig. 2C).

The 2^nd^ power difference in the size dependence of both mechanisms allows us to separate not only particles with different sizes, but also closely similar particles with overlapping distributions (Fig. 2D–H). Particles were isolated at 1.5 mL/min flow rate (Re = 215). High purity of small 4.1 μm particles was obtained at convex side of the channel but at low 12 ± 1% yield (Fig. 2D,E). The small 4.1 μm particles were primarily focused by Dean vortices to the channel center with a yield of 55 ± 5%. Purity of 4.1 μm ranged from 79 ± 1% compared to similarly sized 5.56 μm particles, with overlapping distribution (Fig. 2D,F) to 97 ± 1% when separated from larger 9.95 μm particles (Fig. 2E). The large 5.56 and 9.95 μm particles were primarily pushed toward the concave side of the channel by opposing shear-induced forces^10^. Purity of larger particles ranged from 76 ± 9% to 90 ± 5% for 9.95 μm and 5.56 μm particles, respectively, with similar yields of 60 ± 9%. To demonstrate that similar mechanisms may hold for yeast isolation, we captured a streakline image of calcofluor-stained yeast population. As most yeast in a heterogeneous mixture are young, the streakline coincides with 4.1 μm beads (Fig. 2G). Thus, our device should be able to separate adult yeast (3–10 scars) with a diameter of 5.4 to 11 μm, from young cells (0–2 scars) that are 2.3 to 4.7 μm in diameter with high fidelity (Fig. 2D,E). We note that a flow rate of 1.5 mL/min allows us to isolate 10^7^ yeast/min from standard suspension for bulk metabolomic and proteomic analysis with minimal clogging due to the high pressure applied.

### Microfluidic sorting of yeast

**Figure 3:**
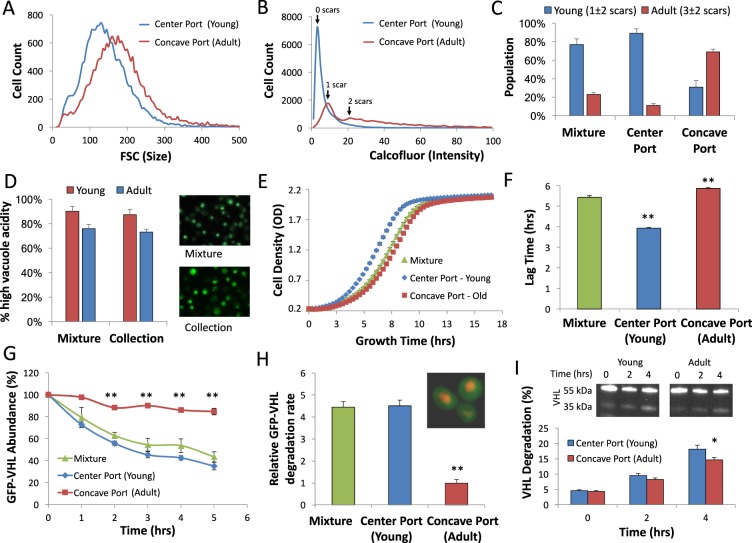
Analysis of isolated age-dependent yeast sub-populations. **(A)** Histogram of forward scatter (FCS) of microfluidic-sorted yeast populations collected from the center and concave ports. Forward scatter is correlated to particle size, 34% shift in FCS shows that yeast collected from the concave port are larger. **(B)** Histogram of calcofluor white intensity of microfluidic-sorted yeast populations. Yeast collected from the center port show minimal staining correlating to 0 to 1 replication scars (blue). Yeast collected from the concave port showed 3-fold higher intensity, correlating with 1 or more replication scar (red). **(C)** Single cell analysis of replicative age distribution in original yeast population (mixture) and cells isolated from the center and concave ports. Cells isolated from center port had an average scar number of 1.3 ± 1.6, and thus are labeled young. Cells from concave port had an average scar number of 3.4 ± 1.7, and thus are labeled adult. **(D)** Reduced vacuolar acidity is an early aging marker^24^. Yeast sorted using our microfluidic device showed similar distribution of vacuolar acidity compared to unsorted mixture (p = 0.452, n = 3), showing that shear stress had no effect on traditional age-related properties of yeast. **(E)** Growth rates of microfluidic sorted yeast populations compared to the original mixture. **(F)** Lag time to exponential stage was 27% shorter in young yeast (0–2 scars) and 8% longer in adult yeast (3–10 scars) compared to the original mixture and adult yeast. **p < 0.01. **(G)** Relative GFP-VHL abundance normalized to NLS-tRFP measured for adult, young and heterogeneous mixture. VHL is a recognized misfolded protein in yeast. Adult cells show a significant delay in GFP-VHL degradation. **p < 0.01. **(H)** Relative GFP-VHL degradation rate was 4.5-fold lower in adult (3–10 scars) yeast than the original mixture or young yeast. In the corner: fluorescent micrograph of yeast expressing GFP-VHL and NLS-tRFP superimposed. **p < 0.01. **(I)** VHL degradation levels measured with western-blot image processing. The degraded proteins increased from 4-fold in the central port collection, while in the concave port collection it increased only by 3.4-fold over the same time period (p = 0.02, n = 3).

Yeast cells suffer minimal damage when exposed to shear stress of up to 1292 Pa^22^ and thus can be separated in high Reynolds flows without damage. Our microfluidic device contains 5 exit ports (Fig. 2H) for simultaneous, parallel collection. Yeast cells were introduced into the device at 1.5 mL/min, experiencing a shear stress of 691 Pa, significantly below their damage threshold. Samples were taken from the central port that should predominantly contain young yeast (0–2 scars) and from the concave port that should be enriched with adult yeast (3–10 scars). FACS measurement of the sorted yeast populations shows 34% increase in forward scattering, a measurement of particle size (Fig. 3A). This result confirms the small but distinct size difference between the sorted populations. Calcofluor white staining of the sorted populations shows 3-fold higher intensity in concave port cells (Fig. 3B). Since the budding scars are the source of the bright fluorescence (Fig. 1A,B), the difference in scar number between the populations results in a large difference in calcofluor fluorescence. Single cell analysis showed that young cells (0–2 scars) were enriched from 77 ± 5% in the mixture to 89 ± 7% in the central port (Fig. 3C), with an average scar number of 1.3 ± 1.6, while adult cells (3–10 scars) were enriched from 23 ± 2% in the mixture to 69 ± 3% in the concave port (Fig. 3C), with an average scar number of 3.4 ± 1.7, suggesting the concave port isolate was primarily adult yeast that underwent at least one replication cycle.

### Age dependent growth rate of the sorted yeast populations

To verify that the subpopulations of yeast cells sorted by microfluidics correlate with familiar age-related properties of yeast^23^, we used single cell analysis to compare vacuolar acidity, whose absence is an early aging marker^24^ in our sorted and unsorted cell populations. We show 16.1 ± 2% lower acidity in adult compared to young yeast sorted using our microfluidic device (Fig. 3D), compared to 15.8 ± 3.4% in the unsorted population (p = 0.452, n = 3), showing that shear stress had no effect on traditional age-related properties of yeast. Similarly, while growth rates in the exponential stage were similar for both populations (Fig. 3E), the lag times were different (Fig. 3E,F). The adult yeast subpopulation taken from the concave port showed a lag time of 5.9 hours, which was 8% higher than the original mixture (p = 0.007). However, the young subpopulation showed a lag time of 3.9 hours, which was 27% lower than the original mixture (p = 0.001). This analysis demonstrated that the size-sorted subpopulations have different, age-related, functional properties similar to traditional assays, leading us to run a comparative analysis of protein degradation in the sorted populations.

### Protein degradation in sorted yeast populations

Proteasome degradation was shown to progressively decrease with aging in both mammals and yeast^25^, and suggested as a mechanism of age-dependent neurodegenerative diseases^26^. Attempts to quantify age-dependent degradation rate of misfolded proteins have thus far been limited to single-cell analysis of hundreds of cells resulting in high variance, and significant differences identified only for old yeast with 6–7 scars (see Fig. 1G). Taking advantage of the high yield of yeast populations sorted by our device, we quantified the age-dependent degradation rate in a high throughput assay. We expressed Von Hippel-Lindau protein (VHL), a spontaneously misfolded protein in yeast, fused to GFP as a tag protein, under the control of GAL inducible promoter. This allowed us to shut down expression of the VHL by providing glucose instead of galactose in the media, and following the degradation rate of the previously accumulated VHL. We normalized the GFP-VHL intensities to a stable NLS-tRFP that is expressed at identical levels under the control of a GAL promoter. Then we isolated 5 samples of 100,000 to 200,000 cells of each subpopulation and ran a neck-to-neck comparison of degradation using FACS (*methods*).

Interestingly, we identified significant differences in misfolded protein degradation between the adult (3.4 ± 1.7 scars) and young (1.3 ± 1.6 scars) yeast populations (Fig. 3G). While GFP-VHL degradation rate was not significantly different between young yeast and the original mixture (Fig. 3H), it was 4.5-fold lower in the adult yeast subpopulation (p = 0.009). We note that 87% of the adult yeast population has 5 scars or less, and that the average scar number in this population was 3.4, suggesting that misfolded protein degradation rates are affected already at early stages of replicative aging in yeast. To verify that the lower GFP measured is a result of the VHL degradation and not a result of GFP degradation, we performed western-blot of VHL using anti-GFP antibodies and quantified the degraded percentage of VHL using image processing. Western-blot analysis shows 29 ± 3% difference in VHL degradation between adult and young cells (p = 0.031, n = 3) confirming that the decrease in GFP measured is a result of VHL degradation (Fig. 3I).

### Mass spectrometry of sorted yeast populations

Our microfluidic approach permits the continuous isolation of large quantities of yeast cells for proteomic analysis, allowing us to identify differences in the yeast proteome at the first steps of replicative aging. Yeast cells were separated into young (1.3 ± 1.6 scars), adult (3.4 ± 1.7 scars) and the original mixture, lysed, trypsin-digested and subjected to system-wide proteomic analysis by LCMS. Samples of each group from two independent experiments were analyzed in triplicates. We identified 932 proteins present in all six sample groups (Table S1). According to the Gene Ontology (GO)-based annotation of cellular localization, the majority of our identified proteins are cytoplasmic (389), and associated with mitochondria (255), roughly half of them (112) are localized to the mitochondrial matrix, others to the organelle membranes (132), vacuole (8), stress granules (9), Golgi vesicles (13), and nucleus (21) (Table S2) in agreement with general distribution of yeast proteins^27^.

**Figure 4:**
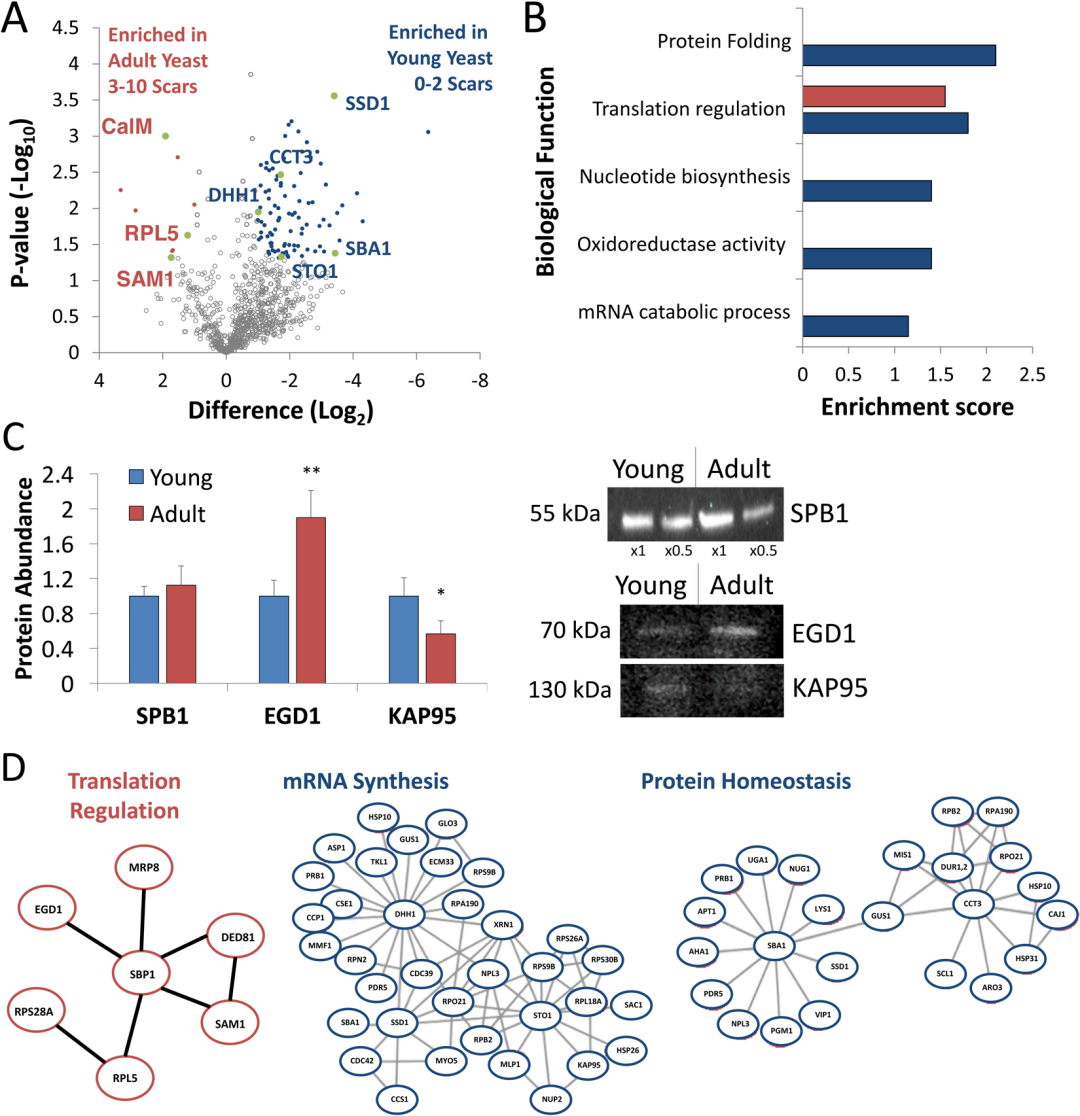
Proteomics analysis of the young and adult yeast cells (**A**) Volcano plot shows the (−log10) Welch t-Test P-value of each protein versus the Welch t-Test difference (log 2) between the two samples. Proteins that changed significantly (FDR = 0.05, S0 = 0.1) by at least two-folds with p-value < 0.05 are colored in blue (upregulated in young cells) or red (upregulated in adult cells). The complete list of the significantly different proteins can be found in Table S4. (**B**) Annotation of significantly different proteins and their enrichment over yeast proteome^51^, showing differences in protein folding and translation. (**C**) Quantitative western blot validation of selected proteins. SPB1 is equally abundant in both populations and its quantity did not change significantly (p = 0.44, n = 3). EGD1 was enriched by 2-folds in the adult population (p = 0.02, n = 3), while KAP95 was enriched by 2-folds in the young population (p = 0.049, n = 3). ×0.5 notes 2-fold dilution of ×1. (**D**) Interactions of significantly different subset of proteins between young and adult cell cultures. Interaction map of upregulated proteins in young culture (blue) and adult cells (red) was derived by using STRING database of interactions and visualized by Cytoscape. To reduce complexity, subnetworks were selected for presentation. On right, subnetworks of proteins upregulated in young cells interacting with chaperones CCT3 and SBA1, and mRNA associated proteins DHH1, SSD1. Central panel presents subnetwork of translation regulators in young cells.

Label-free quantification (LFQ) showed that the majority of the proteins did not change between the populations, with mean standard deviation less than two-fold (Fig. S1), demonstrating the robustness of our analysis. ANOVA identified 64 proteins that were differentially expressed between all three groups (p < 0.05). As expected, the proteomic profile of the original mixed population was more similar to the young than the adult yeast cells (Fig. S1, Table S3). To elucidate differences between populations we used a stringent criterion (FDR = 0.05, p < 0.05) to identify 95 proteins enriched at least 2-fold in the young and 9 proteins enriched in the adult cells (Fig. 4A,B). Enrichment analysis showed that young cells differentially expressed proteins involved in protein homeostasis, including chaperones and co-chaperones. Other enriched proteins included regulators of mRNA metabolism and translation (Fig. 4A,B, Table S4). Western blot analysis validated our findings, showing SPB1 abundance did not change, while EGD1 and KAP95 were enriched (p = 0.04, n = 3) in the adult and young populations, respectively (Fig. 4C).

Analysis of protein-protein interactions showed clustering of proteins differentially expressed in young cells (Fig. 4D, *blue*). One such cluster represents mRNA binding and metabolism associated proteins, where DHH1, STO1 and SSD1 are highly interacting hub proteins, with 18, 14 and 9 connections respectively among the enriched proteins in young cells. Both DHH1 and SSD1 are associated with longevity in yeast^28,29^. Similarly, chaperones CCT3 and SBA1 formed a network with upregulated proteins in young cells, suggesting upregulation of protein folding, and its downregulation after the first few budding events in adult cells (3.4 ± 1.7 scars). CCT3 is a subunit of TRiC which is crucial for VHL recognition and degradation^30^, and its downregulation may be a cause of slow VHL degradation in adult cells (Fig. 3G–I).

Annotation and connectivity analysis of upregulated proteins in the adult cells pointed to a small subset of translation regulatory network where translation regulator SBP1 is a highly connected hub protein. Interestingly, Calmodulin, RPL5 and SAM1 showed significant upregulation in adult cells (Fig. 4A,D, *red*, Table S4). Calmodulin and RPL5 play a significant role in apoptosis and senescence^31,32^ and the deletion of SAM1 extends life cycle in yeast^33^. Thus, our microfluidic separation allowed us to detect a distinct proteomic profile of two extremely close populations of young and adult cells, originating from the same culture, and separated by 2–3 replication events.

## Discussion

The mechanism that regulates senescence is among the most ancient enigmas. While most cellular and organismal systems are thought to decline late in the aging process^34^, recent efforts attempt to analyze the mechanism of aging by identifying discrete aging determinants and the stage in which they appear. *Saccharomyces cerevisiae*, the baker’s  yeast, allows us to dissect the basic mechanisms of aging at high temporal resolution. There are two aspects of aging in yeast: chronological and replicative aging^35^. Chronological aging corresponds to the life span of a non-dividing cell, while replicative aging corresponds to the number of cell divisions that an individual yeast cell goes through. Each cell division is asymmetric, producing a daughter cell that is smaller than the mother cell, with the latter accumulating a bud scar (Fig. 1A). Budding yeast divide asymmetrically with respect to aging as well, resulting in the accumulation of aging factors, such as damaged proteins and unfit organelles by old mother cells (Fig. 1G)^1^.

Microfluidic cell isolation using inertial focusing is an appealing process due to its low cost and continuous operation of the disposable polydimethylsiloxane (PDMS) chip^10,14^. However, current approaches predominantly sort particles in low flow rates, increasing the risk of clogging and resulting cross-contamination. In addition, the low resolution of current devices led to primarily coarse separations of budded yeast from mother-daughter pairs, where significant size difference makes the separation trivial^12^. In contrast, our approach relies on our recently described^14^ opposing shear-induced forces that push large particles towards the concave side of curved channels (Fig. 2A,C). Superimposed on this mechanism are the effects of Dean vortices confining small particles to the center of the channel due to rotation induced lift forces (Fig. 2B,C). As inertial forces scale as a^4^, and Dean vortices induced forces scale as a^2^, our method permits sensitive separation that theoretically scales as a^2^, where a is the particle diameter. High Reynolds that limit clogging and permits the processing of up to 10^7^ cells/min offers a unique tool to separate microbiota for metabolomic and proteomic analysis. We applied this separation mechanism for the challenging sorting of 4.1 μm beads and 5.56 μm beads, reaching purity of 90.6% ± 8% from a heterogeneous mixture. The separation resolution is much higher than previous works that demonstrated separation of cells at least 5 μm in diameter larger between the two sorted populations^10,36^.

Using microfluidic separation we isolated predominantly young (1.3 ± 1.6 scars) and adult (3.4 ± 1.7 scars) yeast from the center and concave ports, respectively, without affecting viability or function (Fig. 2F,G). Surprisingly, even that small difference in replicative age led to significant changes. Young yeast showed a significantly 27% shorter lag time to exponential growth (Fig. 3F), while adult yeast were not significantly different from the original mixture. VHL is a human tumor suppressor that misfolds in yeast^37^, undergoing proteasome degradation^38^. VHL degradation rates were 4.5-fold lower in adult cells possibly due to downregulation of CCT3 which is crucial for VHL recognition^30^. These results suggest that protein quality control is already affected at early stages of replicative aging, explaining the accumulation of HSP^38,39^ during early stages of replicative aging (Fig. 1G).

Our high-Reynolds approach allowed us to isolate sufficient cell quantities for mass spectrometry, showing that young (1.3 ± 1.6 scars) and adult (3.4 ± 1.7 scars) cells, originating from the same culture but separated by 2–3 replication events, have distinct proteomic profiles. We show that changes in classical aging and youthfulness markers such as CalM1, SAM1, RPL5, SSD1, and DHH1 may actually occur earlier than previously reported. Our results support earlier proteomic analysis of age-dependent changes in *Caenorhabditis elegans*, reporting a decline in protein homeostasis in adult nematodes.

In summary, our microfluidic platform offers the ability to rapidly isolate large quantities of microorganisms for proteomic and metabolomic analysis, offering insights into population composition and dynamics. While our current findings could have implications for understanding cellular aging in eukaryotes, the technology can be readily applied to study pathogens, different yeast populations and the diverse human microbiome.

## Methods

### Yeast strains, growth conditions, and materials

Yeast growth, media preparation, and manipulations were performed as previously described^40^. To increase the adult yeast population, cell were grown in high-density cultures in YPD medium for 3 days, and the medium was switched to fresh medium every 24 hours, without diluting the culture. Yeast strain used in this study is BY4741 except for the VHL degradation assay. For the VHL degradation assay, plasmid pESC-URA GAL1p GFP-VHL/GAL10p NLS-TFP (DK5 plasmid)^40^ was used. The VHL gene was fused to GFP and was expressed under the control of a galactose-regulated bi-directional promoter (Gal1p); NLS-TFP was used as a nuclear marker and expressed under Gal10p.

### Microscopy

Yeast cells were grown in liquid medium to mid-Log phase and seeded on concanavalin A (Sigma-Aldrich) coated four-well microscope plates (IBIDI). To label the cell walls of living cells, cells were stained for 30 min at room temperature with rhodamine-concanavalin A (Vector Laboratories) diluted 1:100 from 5 mg/ml stock solutions, and with Calcofluor White (Sigma-Aldrich) diluted 1:10 from 1 mg/ml stock before experiments to stain the bud-scars. The nuclei were tagged with Htb2-GFP from endogenous GFP-tagged yeast library. Confocal 3D images were acquired using a dual point-scanning Nikon A1R-si microscope equipped with a PInano Piezo stage (MCL), using a 60x PlanApo VC oil objective NA 1.40. Calculations of the fluorescence intensity and image processing were performed using NIS-Elements software.

### Single Cell Analysis Algorithm

Single cell analysis of yeast was performed with CellProfiler software^16^. For yeast membranes identification, circular shapes were enhanced in the raw image. Circles ranging from 1.5 to 15 µm (15–150 pixels) diameter were identified using Otsu-Adaptive method and two-class thresholding. Membrane geometrical properties were measured. Threshold correction factor of 2 ± 0.5 was applied to avoid background noise identification as cells. Clumped objects were distinguished by shape. Budding scars and HSP104 foci were identified in a raw image masked by the formerly identified yeast membranes, using Robust-Background-Per-Object method. Threshold correction factor of 1.5 ± 0.5 was applied to determine the identified objects. In all objects identifications, exact threshold for an image series was calibrated using side-by-side examination of 5–10 images. Scars and foci were related to cells using Relate-Objects function. CellProfiler code is available upon request.

### FACS characterization of microfluidic separation

Characterization and assessment of purity and efficiency were performed in the microfluidic device with a microchannel height of 30 µm. Uniformly dyed microspheres with three different diameters: 9.94 µm (Yellow Envy, Bangs Laboratories, Inc., Fishers, IN, USA), 5.56 µm (Suncoast Green, Bangs Laboratories, Inc., Fishers, IN, USA), 4.1 µm (Yellow, Spherotech, Lake Forest, IL, USA) were used to mimic a yeast suspension. Each solution of single type of beads were prepared in deionized water, using beads volume to get an overall same number of beads in each suspension. 3 suspensions of each bead type, and 2 solutions of a mixture (1:1 ratio) of 9.94 µm + 4.1 µm and 5.56 µm + 4.1 µm were prepared for the characterization. Bead suspensions were loaded in a syringe and connected to the input of the channel. Bead suspensions were pushed in the microfluidic device at a flow rate of 1.5 ml/min using the Fusion 200 syringe pump (Chemyx Inc., Stafford, TX USA). Fluorescent images were taken while the suspensions were flowing in the channel to validate the separation. For each suspension separation, output was collected from all 5 ports, in triplicates. Each sample that underwent separation was analyzed by FACS (BD FACSAria^TM^ III, BD Biosciences, San Jose, CA USA). The data was analyzed with FCS Express Flow Cytometer Data Analysis Software (De Novo Software, Glendale, CA USA). Same procedure was established for the fixed yeast suspension stained with Calcofluor white stain to measure the FSC and SSC over the fluorescence.

### Growth assay

Yeast cells were grown overnight, diluted to optical density (OD) of 0.2 measured at 600 nm and sorted by the microfluidic device. Cell density was diluted again to OD_600_ of 0.2 and cells were incubated while shaking in a TECAN Infinite M200 plate reader. Measurements were taken every 20 min for 22 hours. Time dependent OD curves were extrapolated into 9th grade polynomials with MATLAB, and the lag phase time was calculated according to the maximal polynomial 2nd derivative.

### HSP104 aggregation assay

Yeast cells expressing HSP104 endogenously tagged with GFP^41^ were grown as described, without any heat shock, labeled with calcofluor white and imaged. Pictures were analyzed for HSP104-GFP inclusions and scar number using CellProfiler program.

### Protein degradation assay

Cells were grown as described above. The expression of GFP-VHL variants was induced by switching to galactose-containing medium for 3 days. The degradation was calculated as a decrease in the green fluorescence intensity after cells were transferred to glucose medium. The fluorescence intensity of single cells was calculated using FACS. All data points were normalized to GAL induced nuclear localization sequence tag red fluorescence protein (NLS-tRFP) fluorescence decay expressed on the same plasmid.

### Analysis of levels of GFP-fusion proteins and VHL-GFP degradation

Strains, containing GFP-fusion proteins corresponding to hits from the proteomic data were obtained from the GFP-fusion collection. They were then grown and separated in standard conditions. Lysates were obtained using alkaline lysis^41,42^ After SDS-PAGE and transfer to nitrocellulose membrane, the membranes were stained with Ponceau S to obtain data on the amounts of protein in the samples. This was essential, since the amount of cells for analysis was low and protein loading was difficult to equalize by other means. After western blotting with anti-GFP antibodies (ab290 Abcam), the resulting Ponceau S and chemiluminscence images were subjected to densitometry using ImageJ software. Correction for protein loading was performed by dividing the ratio observed in the blotting images, by the ratio calculated for the Ponceau S images. The latter was calculated as the densitometric ratio between the relevant samples, multiplied by 2 for each 1.2 fold difference in Ponceau S ratios. The correction coefficient was calculated from a calibration curve obtained from staining serial dilutions of Ponceau S. The calculated correction factor correlates well with previous results^43^.

Assay for VHL degradation was performed using the W303 strain with four integrated copies of VHL-GFP^44^. VHL-GFP was under the control of a CUP1 promoter and its production was induced by overnight incubation in SD-medium containing 100. VHL-GFP was under the control of a CUP1 promoter and its production was induced by overnight incubation in SD-medium containing 100 µM CuSO4. The chase procedure was done according to Buchanan *et al*.^45^. Quinacrine staining was performed according to Hughes and Gottschling^24^.

### Fluorescence-activated cell sorting analysis

Flow cytometry was carried out on Becton Dickinson FACSAria III. Yeast strains were grown on SC-URA selection medium for 2 days, diluted twice a day to log phase, and on the third day, the fluorescence intensity of the GFP tag fused to VHL and NLS-tRFP was analyzed by fluorescence-activated cell sorting with a 488 and 561 laser using BD FACSDiva (BD Biosciences) software.

### Statistics for image processing, VHL degradation and lag time

The experiments were repeated at least 3 times. Multiple correlation coefficients were calculated for a single variable in the analyzed group, with subsequent regression analysis to determine p-values. Standard comparisons were performed using t-Test.

### Mass spectrometry

Mass spectrometry of yeast cells was carried out as previously described^46^.

#### Sample preparation for MS analysis

Pellet of 10^7^ yeast cells was re-suspended in 0.1 M NaOH and incubated 5 min at room temperature. After washing out NaOH, the cells were lysed in buffer containing 100 mM Tris, 2% SDS, and 10 mM DTT for 60 min in room temperature. Lysed samples were prepared using the FASP protocol^47^. Briefly, lysate was transferred to microcon centrifugal filters, Amicon-ultra-0.5 tubes, MW cutoff of 10 kDa (Merk Millipore, Germany) for reduction, alkylation, washing and trypsin digestion. The protein lysate was diluted and washed by denaturation buffer containing 8 M urea in 0.1 M Tris-HCL set at pH 8.5. Proteins were reduced with 10 mM of DTT for 1 hour and then alkylated with 50 mM iodoacetamide supplemented to the denaturation buffer for 1 hour in dark. To remove denaturants and alkylation reagents the proteins were washed five times with the denaturation buffer. Then the protein were digested with 0.4 μg Trypsin (sequencing grade modified trypsin V5111, Promega, Madison, WI) in digestion buffer (10% ACN, 1.3 M Urea, 25 mM Tris pH 8) overnight at 37 degrees. To remove excess of Urea, the peptides were washed with 10% ACN, 25 mM Tris pH 8. The tryptic peptides were eluted from the Amicon tubes by centrifugation at 12,000 g for 10 min, evaporated till dryness, and resuspended in 0.1% formic acid (FA). The peptide mixture was desalted using in-house packed C18 StageTips^48^. The StageTips were conditioned at first with 100% Methanol and then with 80% acetonitrile (ACN). The equilibration was performed with 2 × 0.1 ml 0.1% FA before application of the samples at a total amount of 15 µg total protein per stage tip. The bound peptides were washed with 2 × 0.1 ml 0.1% FA and eluted with 120 µl 80% ACN plus 0.1% FA. The eluted fractions were dried under vacuum and resuspended in 0.1% FA to a final concentration of around 0.4 µg/µl.

#### Nano-LC-MS/MS Analysis

The peptides were injected into a Nano Trap Column, 100 μm i.d. × 2 cm, packed with Acclaim PepMap100 C18, 5 μm, 100 Å (Thermo Scientific) for 8 min at flow 5 ul/min, and then separated on a C18 reverse-phase column coupled to the Nano electrospray, EASY-spray (PepMap, 75 mm × 50 cm, Thermo Scientific) at flow 300 nl/min using an Dionex Nano-HPLC system (Thermo Scientific) coupled online to Orbitrap Mass spectrometer, Q Extactive Plus (Thermo Scientific). To separate the peptides, the column was applied with a linear gradient with a flow rate of 300 nl/min at 35 °C: from 1 to 25% in 82 min, from 25 to 35% in 45 min, from 35 to 55% in 43 min, from 55 to 90% in 5 min, and held at 90% for an additional 30 min, and then equilibrated at 1% for 20 min (solvent A is 0.1% formic acid, and solvent B is 80% acetonitrile, 0.1% formic acid). The Q Exactive was operated in a data-dependent mode. The survey scan range was set to 200 to 2000 m/z, with a resolution of 70,000 at m/z. Up to the 12 most abundant isotope patterns with a charge of ≥2 and less than 7 were subjected to higher-energy collisional dissociation with a normalized collision energy of 28, an isolation window of 1.5 m/z, and a resolution of 17,500 at m/z. To limit repeated sequencing, dynamic exclusion of sequenced peptides was set to 60 s. Thresholds for ion injection time and ion target value were set to 70 ms and 3 × 10^6^ for the survey scans and to 70 ms and 10^5^ for the MS/MS scans. Only ions with “peptide preferable” profile were analyzed for MS/MS. Data was acquired using Xcalibur software (Thermo Scientific).

#### Data Analysis and Statistics

For protein identification and quantification, we used MaxQuant software^49^, version 1.5.3.30. We used Andreomeda search incorporated into MaxQuant to search MS/MS spectra against the UniProtKB database of Saccharomyces cerevisiae proteome, (Uniprot release, Aug 2015). The identification allowed two missed cleavages. Enzyme specificity was set to trypsin allowing cleavage N-terminal to proline and up to two miscleavages. Peptides had to have a minimum length of seven amino acids to be considered for identification. Carbamidomethylation was set as a fixed modification, and methionine oxidation was set as variable modifications. A false discovery rate (FDR) of 0.05 was applied at the peptide and protein levels. Initial precursor mass deviation of up to 4.5 ppm and fragment mass deviation up to 20 ppm were allowed. Only proteins identified by more than 2 peptides were considered. To quantify changes in protein expression we used the label-free quantification (LFQ) using the MaxQuant default parameters^49^. For statistical and bioinformatic analysis, as well as for visualization, we used the Perseus software^50^. (http://www.biochem.mpg.de/5111810/perseus). For annotation, the DAVID webserver^51^ was used. STRING server (http://string-db.org/)^52^ was used to define interacting proteins, which were visualized by using Cytoscape software^53^.

### Device fabrication

Serpentine shape molds were fabricated by photolithography of SU8 on silicon wafers. Channels were replica molded in PDMS and bonded to glass using oxygen plasma as previously described^10,14^. Channels and glass were cleaned using M3 low-residue adhesion tape. This cleaning made acid processing redundant and enabled device durability to velocities up to 12 m/s. High aspect ratio channels were designed to achieve distinct horizontal focusing locations. Channels are composed of four half-circle arcs each of them is 33 μm high, 200 μm wide, and the radius of curvature is 900 μm. Prior to the experiment the channel was coated with Pluronic F-68 (Sigma-Aldrich) for 1 hour at room temperature to prevent non-specific adhesion of fluorescent particles.

### Yeast sorting

Yeast cells were grown as described, pelleted by centrifugation and re-suspended in water. Cells density diluted to OD of 0.2 and injected to the microfluidic device at 1500 μl/min. The high fluid velocity enables binning directly from a wide cone shaped drainage section (Fig. 2D,E) avoiding the risks of particle sedimentation and division sub-channels blocking.

## Electronic supplementary material

Supplementary information

Supplementary Tables

Supplementary Table

Supplementary Table
