# Supplementary material for: High-Reynolds Microfluidic Sorting of Large Yeast Populations
Source: Sci Rep. 2018 Sep 13;8:13739. doi: 10.1038/s41598-018-31726-6 (PMC6137188; doi:10.1038/s41598-018-31726-6)
Supplement: Supplementary file 1 — Supplementary information [file 41598_2018_31726_MOESM1_ESM.pdf]

High-Reynolds Microfluidic Sorting of Large Yeast Populations

Eliezer Keinan[1], Ayelet Chen Abraham[2], Aaron Cohen[1], Alexander I. Alexandrov[2,4], Reshef Mintz[3], Merav Cohen[1,2], Dana Reichmann[3], Daniel Kaganovich[2, *], Yaakov Nahmias[1,2,*]

1. *Alexander Grass Center for Bioengineering, Benin School of Computer Science and Engineering, The Hebrew University of Jerusalem, Jerusalem, ISRAEL*
2. *Department of Cell and Developmental Biology, Silberman Institute of Life Sciences, The Hebrew University of Jerusalem, Jerusalem, ISRAEL*
3. *Department of Biological Chemistry, Silberman Institute of Life Sciences, The Hebrew University of Jerusalem, Jerusalem, ISRAEL*
4. *Current address: Fundamentals of Biotechnology Federal Research Center of the Russian Academy of Sciences, Bach Institute of Biochemistry RAS*

* Correspondence should be addressed to:
Yaakov Nahmias: ynahmias@cs.huji.ac.il
Daniel Kaganovich: dan@cc.huji.ac.il

Supporting Information

Figure S1. Proteomic analysis of three sample sets, young, adult and mix. **(A)** clustering analysis of all 932 proteins identified in this analysis. Clustering done on median LFQ intensity values using the Perseus software[52]. **(B)** Clustering analysis of significantly different proteins identified by the ANOVA test (P-value <0.05). The LFQ values were normalized using z-scoring ranging from -2 (low LFQ intensity) to 1.8 (high LFQ intensity).

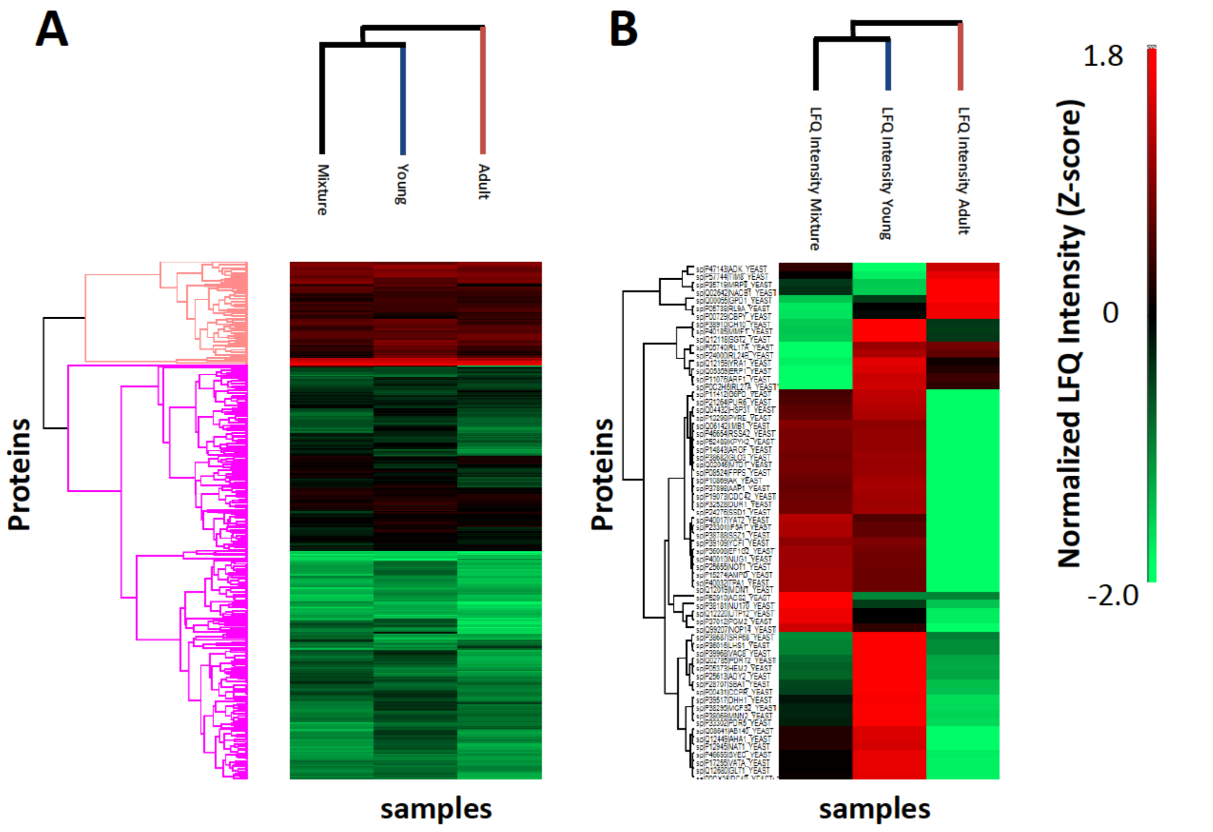

Table S1. Proteins which are present in all yeast groups: young (0-2 scars), adult (3-10 scars) and heterogeneous mixture.

Table S2. The cellular distribution of the proteins according to GO based cellular localization.

Table S3. ANOVA test which identifies a subset of 64 proteins showing significantly different expression levels between all three yeast groups: young (0-2 scars), adult (3-10 scars) and heterogeneous mixture.

Table S4. Distribution of biological functions within the group of significantly different proteins.
